# The sweet spot study—Developing e-liquid product standards for nicotine form and concentration to improve public health: Protocol for a randomized, double-blinded, crossover study

**DOI:** 10.1371/journal.pone.0291522

**Published:** 2023-09-12

**Authors:** Yoo Jin Cho, Marielle C. Brinkman, Alice Hinton, Jean D. Nshimiyimana, Toral Mehta, Ayomipo Adeniji, Kaila Norton, Ahmad El Hellani, Theodore L. Wagener

**Affiliations:** 1 Center for Tobacco Research, The Ohio State University Comprehensive Cancer Center, Columbus, OH, United States of America; 2 Division of Epidemiology, College of Public Health, The Ohio State University, Columbus, OH, United States of America; 3 Division of Biostatistics, College of Public Health, The Ohio State University, Columbus, OH, United States of America; 4 Division of Environmental Health Sciences, College of Public Health, The Ohio State University, Columbus, OH, United States of America; 5 Division of Medical Oncology, Department of Internal Medicine, The Ohio State University Wexner Medical Center, Columbus, OH, United States of America; Federal University of Juiz de Fora, BRAZIL

## Abstract

**Objectives:**

E-cigarettes pose significant risks to youth, but smokers may benefit from switching to e-cigarettes by reducing their exposure to toxicants, which creates a challenge for the Food and Drug Administration (FDA) in regulating e-cigarettes to protect population health. This study aims to develop e-liquid product standards for nicotine form and concentration that reduce the appeal of e-cigarettes to young people while keeping e-cigarettes available as a safer alternative for smokers.

**Design and participants:**

A single-visit, double-blinded, randomized crossover design will be used to examine the effects of e-liquids with varying fractions of free-base nicotine (5%, 25%, 45%, 65%, 85%) among a sample of 66 young adult EC users and 66 older adult smokers, across ecologically valid total nicotine concentrations (20 mg or 50 mg/mL).

**Interventions and outcomes:**

A 2-puff session will be conducted to test each of the 10 e-liquids in randomly assigned sequences, followed by a 10-minute washout period and participant ratings on appeal and sensory attributes such as throat hit and harshness, as well as behavioral intentions for continued use. Generalized linear mixed models will be used to determine a free-base nicotine level that has limited or no appeal to young adult e-cigarette users while remaining acceptable to smokers.

**Conclusions:**

This study will provide the FDA with scientific evidence regarding the effect of product standards that mandate a minimum threshold for the fraction of free-base nicotine.

**Trial registration:**

The study is registered on clinicaltrials.gov under the identifier NCT05864586.

## Introduction

Regulating e-cigarettes (ECs) is challenging. While ECs pose significant risks to young people by increasing the risk of nicotine initiation and sustained nicotine addiction [1] and use of more harmful tobacco products [2, 3], current smokers may benefit from completely switching to ECs by reducing their exposure to toxicants [4, 5]. The US Food and Drug Administration’s (FDA) regulation of ECs must be based on evidence demonstrating they are “appropriate for the protection of public health [6].” This means FDA must base regulations on evidence of their potential impact on young non-users *and* tobacco users. Viewing ECs through this lens, the FDA needs robust evidence demonstrating how to regulate ECs based on these two competing goals that must be aligned: 1) allowing ECs to remain on the market as appealing, potentially less harmful substitutes for current smokers, and 2) reducing their appeal and addictiveness for young people.

The US Congress and FDA have already implemented several regulatory efforts to curb youth EC use, including: increasing the federal minimum age requirements to purchase tobacco products (including ECs) to 21 years [7]; banning the sale of all flavored cartridge-based ECs [8]; and enforcing its requirements for Premarket Tobacco Product Applications (PMTAs) for ECs, leading to the removal of more than 1 million EC products from the US market (almost all were flavored ECs) [9]. The long-term impact of these regulations on EC use in young people is yet to be determined [4]. While there is strong reason to believe that flavors contribute to EC use in young people [10, 11], flavors do not appear to be the major catalyst for the recent surge in youth EC use. Flavored ECs were on the market since the late 2000s, yet youth EC use declined by 29% from 2015 to 2016 and remained at those levels through 2017 [12]. Youth use skyrocketed only after EC manufacturers manipulated nicotine form to reduce the harshness of the aerosol by reducing the fraction of unprotonated ‘free-base’ (FB) nicotine, which is more prevalent in basic conditions, and increasing the fraction of protonated ‘nicotine salts’ (NS) with acidic additives in the EC liquid. This manipulation made it easier for ‘tobacco-naive’ users to inhale high levels of nicotine, resulting in a significant increase in youth EC use to record highs (a 288% increase from 2017 to 2019) [1, 12–16]. Similarly, the tobacco industry manipulated the pH of cigarette smoke to improve nicotine delivery and palatability of cigarettes, thereby increasing appeal among young people and non-users [17, 18]. Arguably, regulating total nicotine content in ECs may be a more effective regulatory strategy to protect youth, with minimal impact on adult smokers switching to potentially less harmful ECs.

To protect young people and still offer smokers a potentially less harmful alternative, the FDA could establish an e-liquid nicotine product standard that sets a minimum level of FB nicotine content that is unappealing/minimally appealing to young people and non-users but still satisfies nicotine craving in adult smokers. Recent evidence suggests that young exclusive EC users prefer “smoother” e-liquids with very low levels of FB nicotine, but smokers and dual users prefer some level of throat irritation [19], or what the industry has termed “throat hit [20],” that arises from higher levels of FB nicotine in e-liquids. However, to date, no study has systematically examined differing levels of FB nicotine in e-liquids to determine an appropriate minimum level, nor how this level may be influenced by differing levels of nicotine concentrations.

Therefore, the purpose of the present study is to determine this minimum FB nicotine level (5, 25, 45, 65, 85%) across two ecologically valid nicotine concentrations (20 mg/mL and 50 mg/mL) among a sample of young adult EC users with minimal/no history of smoking as well as older adult smokers. We hypothesize that with increasing FB levels (i.e., decreasing NS), ECs will be too harsh for exclusive young adult EC users but not for adult smokers, especially at the higher nicotine concentration.

## Materials and methods

### Design

**Fig 1:**
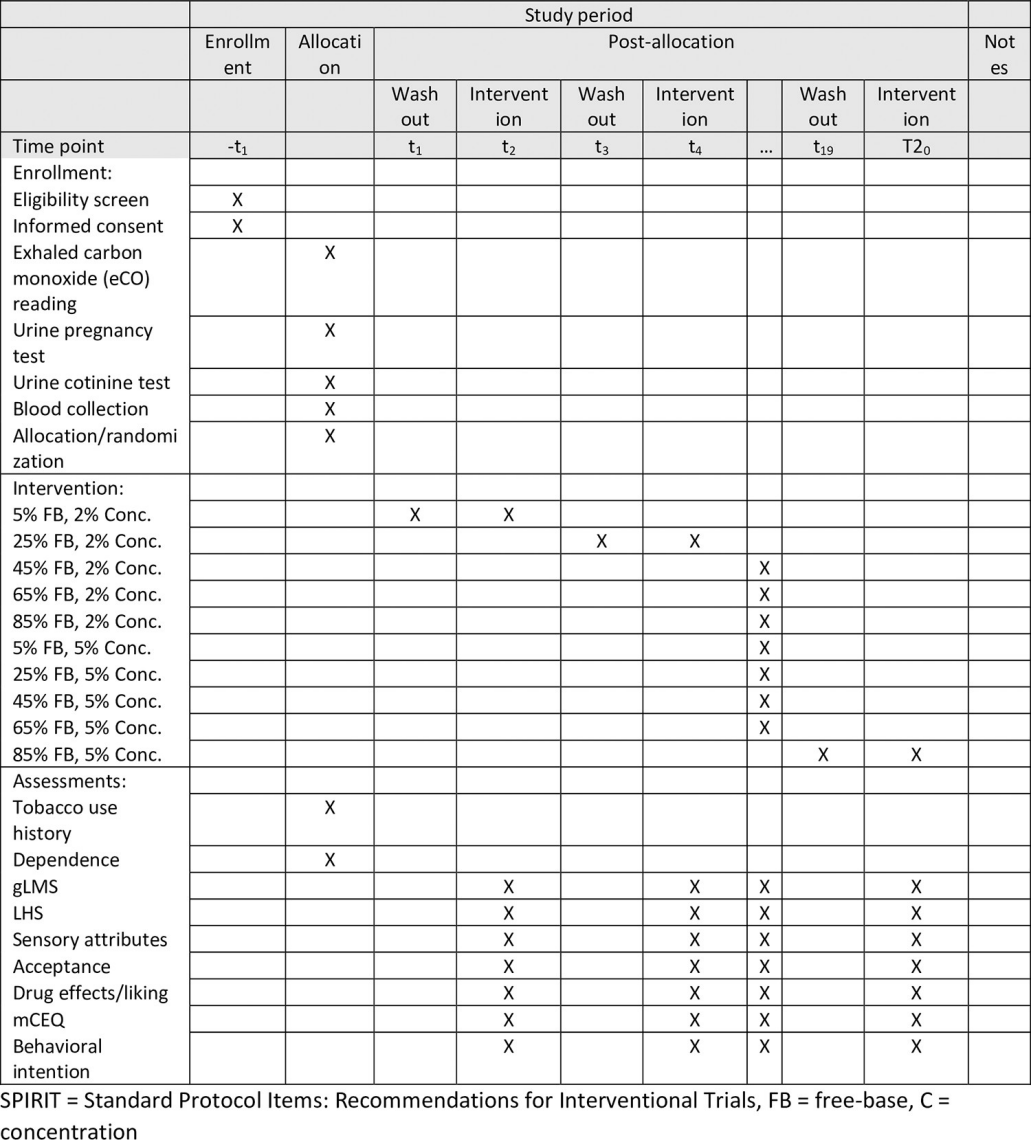
Schedule of enrollment, interventions, and assessments (SPIRIT).

Using a single-visit, double-blind, randomized crossover trial design the study will examine the effects of e-liquids with varying levels of FB nicotine (5, 25, 45, 65, 85%) at two nicotine concentrations (20 or 50 mg/mL) on appeal among young adult EC users with little or no history of smoking (n = 66) and older adult smokers (n = 66). The schedule of enrollment, interventions, and assessments is shown in Fig 1. A 2-puff session will be conducted to test each of the 10 conditions, followed by ratings of appeal and sensory attributes during a 10-minute washout period between conditions. A similar study design has been used to examine the differential appeal of EC flavors, nicotine levels, and the interactive effects between flavor and nicotine [19, 21–23]. The study has been applied for registration at clinicaltrials.gov with an NCT number NCT05864586. The authors confirm that all ongoing and related trials for this intervention are registered.

### Setting

Study visits will take place in the Ohio State University Comprehensive Cancer Center (OSUCCC)’s Center for Tobacco Research (CTR) in Columbus, Ohio. All study visits will be conducted in smoking/vaping rooms under negative pressure.

### Materials

The study team will prepare 10 tobacco-flavored e-liquids, which vary in FB nicotine (5, 25, 45, 65, and 85%) and concentration (20 or 50 mg/mL) and contain commercially available tobacco flavorings, benzoic acid, glycerol (vegetable derived), and propylene glycol (see Table 1). The FB fractions were chosen to cover a wide range, with the lower end being comparable to JUUL and disposable pod-based ECs that are popular among young people [24], and the higher end being comparable to early ECs that contained nicotine mainly in the FB form and were less appealing to young people. The team submitted an Investigational Tobacco Product (ITP) application to the FDA, outlining the e-liquid preparation protocol. Study staff who are not involved with data collection will be responsible for the preparation of the e-liquids. The 10 e-liquids will be labeled with an “investigational use only” descriptor and loaded into commercially available refillable pods (W01 brand’s 1.80 Ohm JUUL battery compatible refillable pods, OVNS, Shenzhen, China) to be vaped in random order by participants using a commercially available EC device (JUUL, San Francisco, USA).

**Table 1:** Ingredients of the investigational tobacco products.

ITP Name	Nicotine Form	–(-)Nicotine (mg/g)	Flavoring	Benzoic acid (mg/g)	Glycerol (mg/g)	Propylene Glycol (mg/g)
5FB20	5% Free-base	20	Tobacco	14.40	676	290
25FB20	25% Free-base			11.37	678	291
45FB20	45% Free-base			8.34	680	292
65FB20	65% Free-base			5.31	682	292
85FB20	85% Free-base			2.27	684	293
5FB50	5% Free-base	50		36.00	640	274
25FB50	25% Free-base			28.42	645	277
45FB50	45% Free-base			20.84	651	279
65FB50	65% Free-base			13.26	656	281
85FB50	85% Free-base			5.69	661	283

### Sample

The study is planned to enroll participants from June 1, 2023 to December 31, 2023. This is a one-time visit study, and there will be no follow-up required. A sample of healthy, young adults who exclusively use ECs and have no or minimal smoking history (n = 66) and older adults (25–50 years) who smoke (n = 66) will be recruited for the study. Participants will be recruited from the general public, using targeted online and social media advertisements, flyers, and word-of-mouth marketing in the Columbus metropolitan area. Various online and social media platforms will be used to distribute advertisements. Participants will be screened initially through an online screener linked to our study advertisement, then over the telephone. Those meeting the following eligibility criteria will be asked to participate and will be compensated for their time ($125/visit). Inclusion criteria: 1) current exclusive young adult EC user (daily use) for at least the past 3 months (confirmed by urinary cotinine and 4-(methylnitrosamino)-1-(3-pyridyl)-1-butanol [NNAL]) between 21–24 years old inclusive, with no or minimal history of smoking cigarettes (<10 cigarettes in lifetime), or current older adult smoker (daily use and ≥100 cigarettes in lifetime) between 25–50 years old inclusive, with interest in trying an EC; 2) willing to abstain from all nicotine product use for 12 hours prior to the study visits; and 3) read and speak English. Exclusion criteria: 1) currently attempting to quit nicotine or tobacco products; 2) currently pregnant (will be verified with a urine pregnancy test), planning to become pregnant, or breastfeeding; 3) current or past use of tobacco products other than ECs or cigarettes (use of ≥ 10 traditional cigars, cigarillos, or filtered cigars in entire life; smokeless tobacco product use for ≥ 10 times in entire life; hookah use in the last 30 days); 4) self-reported diagnosis of lung disease including asthma, cystic fibrosis, or chronic obstructive pulmonary disease; 5) self-reported new or unstable cardiovascular disease diagnosed within the past 12 months.

### Procedures

**Fig 2:**
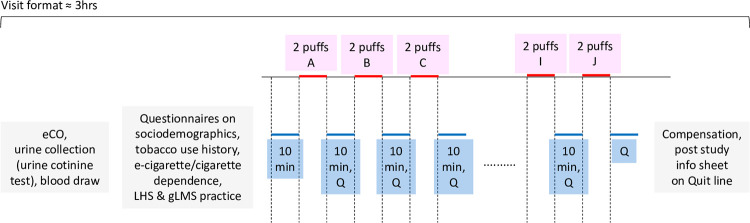
Details of study visit. The 10 e-liquids (A-J) will be provided to participants in random order. LHS = Labeled Hedonic Scale and gLMS = General Labeled Magnitude Scale. “Q” refers to a questionnaire on sensory attributes using gLMS and LHS, overall acceptance, Drug Effects/Liking Questionnaire, the modified Cigarette Evaluation Questionnaire (mCEQ), and behavioral intentions for continued use.

Each participant will complete a single, solo laboratory visit for a maximum of 3 hours (see Fig 2). Participants will be instructed to abstain from nicotine for 12 hours prior to their appointment and will get a reminder the evening before. During the lab visit, participants will first provide informed consent and biospecimens will be collected to confirm pregnancy status, tobacco use status, and 12-hour nicotine abstinence. Participants’ self-reported use of tobacco/nicotine will be confirmed using a testing kit that detects the presence of cotinine (a metabolite of nicotine) in urine (e.g., AimStep® Cotinine Test, which detects urinary cotinine at a concentration of 200 ng/mL). Exclusive EC use status will be verified via self-report and confirmed following post hoc analysis (after the study visit) of urinary NNAL (<0.15 pmol/mL), a metabolite of 4-(methylnitrosamino)- 1-(3-pyridyl)-1-butanone (NNK), that is found in cigarette smokers but is present at an extremely low level in EC users [25]. Cigarette smoking status will be verified via self-report and confirmed following post hoc analysis of urinary NNAL (≥0.15 pmol/mL). Twelve-hour nicotine abstinence will be confirmed using a semi-bogus pipeline [26]. Participants will be informed that their blood plasma nicotine will be analyzed to confirm their abstinence from nicotine, at the time of the visit; however, venous blood will not be analyzed in real-time, but after study visits. Participants with baseline plasma nicotine levels greater than 3 ng/mL will be replaced post hoc with new participants.

After participants complete questionnaires on sociodemographics, tobacco use history, and EC and cigarette dependence, 10 vaping sessions will take place. In each vaping session, participants will inhale two puffs from the study EC device, loaded with one of the 10 research conditions (5 freebase nicotine fractions x 2 nicotine concentrations). Participants will be told to inhale for at least 1 second per puff, while other puffing parameters (i.e., flow rate, maximum puff length, puff volume, and exhalation rate) will not be controlled. Pre-filled and weighed e-liquid pods will be provided to participants in random order. The assigned randomization sequences of the study e-liquids will be generated by a statistician using SAS 9.4. Permuted block randomization, stratified by use (young adult EC users vs older adult smokers), will be employed to ensure that each research condition is sampled an equal number of times in each position. Study staff enrolling participants and collecting data will remain blinded to each participant’s assigned study e-liquids throughout the course of the study.

There will be a 10-minute washout period between conditions to minimize carryover effects, where participants will be instructed to rinse their mouth and spit at least three times with room-temperature water [21]. The washout period stems from the fact that plasma nicotine levels return to baseline 40 minutes after a 10-puff vaping session [27], suggesting that 8 minutes will be sufficient for nicotine levels to return to baseline after two puffs. After each condition, participants will complete questionnaires on the outcome measures outlined below.

### Outcome measures

#### Primary outcome measures

The primary outcome measures are behavioral intentions for continued use, the intensity of sensory attributes, and the degree of liking or disliking of each e-liquid. *Behavioral Intentions for continued use* will be assessed by asking participants how likely they are to: 1) try the product again, 2) purchase the product for regular use, and 3) use the product regularly if this were the only e-cigarette available. Response options will range from 1 “extremely unlikely” to 7 “extremely likely” [28]. The intensity of sensory attributes will be assessed by asking participants to rate the intensity of: 1) throat hit, 2) smoothness, 3) harshness, 4) sweetness, and 5) bitterness of each e-cigarette on the General Labeled Magnitude Scale (gLMS) [23, 29]. Participants will mark their ratings on a vertical line with verbal descriptors ranging from “no sensation” to “strongest imaginable,” and the responses will be coded from 0 to 100 for the analysis. The degree of liking or disliking of each e-liquid will be measured by asking participants to rate how much they like or dislike each e-cigarette using Labeled Hedonic Scale (LHS) [30] with a vertical line with verbal descriptors ranging from -100 “most disliked imaginable” to 100 “most liked imaginable.”

#### Secondary outcome measures

The secondary outcome measures include the subjective appeal and overall acceptance of each e-liquid. Subjective appeal will be assessed using the Adapted Drug Effects/Liking Questionnaire [31] and the modified Cigarette Evaluation Questionnaire (mCEQ) [32]. The Drug Effects/Liking Questionnaire will assess the extent to which participants currently feel the product effect on a scale from 1 to 5 using ten items (e.g., “How strong is your desire or urge to use the study product right now, just for pleasure?”). The mCEQ will assess the degree to which participants experience the reinforcing effects of vaping using 10 items (e.g., “Was vaping the study e-cigarette satisfying?”). The overall acceptance measured on a tobacco industry-designed thermometer scale (from 0 “the very worst” to 100 “the very best”, with 50 indicating “indifferent”) [33].

### Analysis

Statistical analysis will be conducted using SAS 9.4 (SAS Institute, Cary, NC). Participant demographic and other characteristics will be summarized by the study arm. Continuous variables will be reported as mean ± standard deviation and compared using t-tests, whilst categorical variables will be reported as counts and proportions and compared using chi-squared tests. Transformations will be applied to normalize the distribution and stabilize the residual variance where appropriate.

To determine a nicotine fraction limit that is satisfying to smokers while minimally appealing to young adult EC users, generalized linear mixed models will be used to evaluate appeal (e.g., industry thermometer rating and sensory appeal metrics), which include demographic and smoking history as covariates, random subject effects, main effects for usage group (smokers versus EC users), FB fraction (numerical) and concentration (categorical), and interactions between usage group and each of the condition parameter variables (i.e., FB fraction and concentration). A significant interaction between the usage group and a condition parameter variable would suggest that the variable influences the two user groups differently with respect to their appeal. Interactions between condition parameter variables will also be explored. Various link functions and distributions will be employed to complement the data structures for each of the outcomes, including ordinal logistic regression.

Given our goal to find combinations of the condition parameter that are sufficiently satisfying for smokers but do not appeal to young adult EC users, condition combinations will also be directly compared between study arms to identify the condition parameter combinations that result in the greatest difference in appeal between the two user groups. These comparisons will be made using t-tests or Wilcoxon rank-sum tests as appropriate for the structure of the data. Lastly, carryover effects will be explored by estimating the interaction between a given condition and its preceding treatment, and period effects will be examined by testing the interaction between treatment and order. A significance level of p = 0.01 will be used, given the various measurements tested.

### Sample size

A total of 132 participants will be recruited, including young adult e-cigarette (EC) users with minimal or no history of smoking (n = 66) and older adult smokers (n = 66). Conservatively, 10% of 132 participants are assumed to need to be replaced post hoc with another participant due to failing to abstain from nicotine use prior to the study visit. With 120 participants, a correlation of 0.5, and an alpha of 0.01, a two-sided paired t-test has 80% power to detect an effect size of 0.32 standard deviations of the measurement for the concentration main effect. For the FB fraction main effect, the model has 80% power to identify a slope of 0.31 with 120 participants with an alpha value of 0.01. The effect sizes are conservative, given that a previous study [19], which investigated the impact of nicotine formulation on appeal and sensory attributes, observed relatively larger effect sizes, with Cohen’s d values ranging from 0.53 to 1.20. For categorical interactions, the power is estimated by conducting pairwise comparisons of the two interacting groups (a total of 4 groups with two 2-level variables) and adjusting the p-value to 0.0017, accounting for the 6 possible pairwise comparisons (0.01/6). With 30 samples per group, A two-sided, two-sample t-test has over 80% power to detect a 1.7-fold difference between any of the two groups in the interaction term, considering an alpha value of 0.0017 and coefficient of variation value of 0.5. For interactions with FB fraction, group sizes of 60 have 80% power to detect a difference in slopes of 0.43, assuming alpha = 0.01.

### Data management

Study data will be collected, managed, and secured using REDCap, an electronic data collection tool hosted at the OSUCCC, which will be accessible to authorized study personnel who have the necessary password(s). Each participant will be granted a unique identifying number for the study (study ID) and only the research ID will be used to identify all study data. Tablets provided by OSU will be used to enter electronic data (e.g., questionnaire data and laboratory data), which will be collected directly from participants. All computer systems will be password-protected to prevent unauthorized access, and all network-based inter-site communications of confidential data will be encrypted. Access to information stored on a computer will require concurrent knowledge of the data format, computer language, file name, and passwords. Participant information will be accessible only to research staff who are pledged to confidentiality and have received training in the ethical conduct of research (i.e., Collaborative Institutional Training Institute [CITI]’s Human Subjects Protection [HSP] and Responsible Conduct of Research [RCR] trainings). Physical materials will be stored in a locked file cabinet, office, or suite at the CTR.

### Safety considerations

#### Privacy

Study visits will take place in a secure laboratory setting at the CTR where access is limited to study personnel, and researchers will work with only one participant at a time to protect participants’ privacy. Since participants may perceive questions on EC use as intrusive, participants will be informed of the nature of questions in advance.

#### Potential risks

Since current EC users and adult smokers will be recruited, the participants are already smoking or using ECs and will only be asked to use a fully characterized EC device and e-liquids for which an FDA ITP was submitted and will be "approved" (i.e., receive a "no concern" letter from the FDA). The risk of side effects and adverse events associated with the use of ECs is quite low. ECs are commercially available in the US without a prescription. Lab studies of toxicant exposure suggest that ECs incur no greater risk to health than combustible cigarettes. In general, ECs have lower levels of harmful and potentially harmful constituents than cigarettes but are not 100% safe [4]. To date, EC studies describing adverse events report mild and tolerable side effects, including mouth/throat irritation, cough, and headache, which typically resolved completely with continued use. In addition to the previously reported adverse events, the participants have a potential risk of using FB or protonated nicotine more than they have experienced, although this risk will be mitigated as participants are instructed to take only two puffs from a single condition, followed by a 10-minute washout period. The study procedures, including questionnaires, exhaled breath, and urine collection are noninvasive and pose minimal risk to study participants. The risks associated with drawing blood from a vein may include, but are not limited to, brief discomfort at the site of the blood draw, possible bruising, redness, and swelling around the site, bleeding at the site, dizziness, or lightheadedness when the blood is drawn, nausea, and, in rare cases, an infection at the site of the blood draw.

#### Protections against risk

During the study period, all participants will be screened for general medical precautions (e.g., pregnancy, cardiovascular disease) and monitored for adverse events. Any serious adverse events will be reported to the study MPI and subsequently to the Ohio State University Institutional Review Board (IRB). Participants who experience a serious adverse event or have a cardiovascular or pulmonary event will be withdrawn from participation. Blood will be drawn by a trained nurse; only sterile tools will be used, and the participant’s skin will be cleansed with an alcohol wipe at the location of the needle stick. Following the completion of the study, we will encourage participants to quit smoking and/or stop using ECs. Participants will be provided with a post-study information sheet, which includes information about the Ohio Tobacco Quit Line service that offers nicotine replacement therapy resources.

### Ethical declarations

This study has been registered with clinicaltrials.gov under the identifier NCT05864586. This study was reviewed and approved by the Ohio State University Cancer IRB under Study Number 2022C0207 on 4/10/2023. Oral and written explanations of the study and REDCap will be utilized for receiving informed consent and a legally valid signature. During the consent procedure, the voluntary nature of the study and the participant’s right to withdraw at any time will be emphasized, and a written copy of the informed consent will be given to the participant at the moment of consent for them to keep. Personnel approved by the IRB will collect informed consent. Any protocol revisions will be reported to the OSU Cancer IRB and ClinicalTrials.gov and will be noted in manuscripts.

### Trial status

The study is planned to enroll participants from May 2023 to December 2023.

## Discussion

There is an urgent public health need to prevent young people from initiating EC use. However, the potential of ECs to help current smokers quit smoking makes their regulation challenging. While regulatory efforts have been implemented to curb youth use, including banning the sale of flavored cartridge-based ECs, there is a need to regulate nicotine dimensions that make youth users dependent on ECs. This study will help the FDA establish e-liquid nicotine product standards that minimize the appeal of ECs to youth while satisfying adult smokers’ nicotine craving by examining the impact of nicotine form and concentration on sensory perceptions and EC liking.

There are limitations of the study to be acknowledged. First, there is a risk of carryover effect because participants will test 10 e-liquids in a single laboratory visit although a 10-minute washout period will follow sampling each e-liquid to minimize the potential carryover effects. Carryover effects will be explored by estimating the interaction of a given condition with the preceding condition. Also, the 10 test liquids will all be tobacco-flavored, thus flavor effects cannot be assessed. This design was chosen to minimize participant burden and based on the fact that the impact of nicotine concentration on sensory experiences was consistent across different flavors [21]. Still, the study will support future studies aimed to develop e-liquid product standards on EC nicotine dimensions other than concentration and form (i.e., nicotine isomer), as well as the interaction between EC nicotine dimensions and flavoring and their long-term behavioral effects.

Strengths of the study include the use of standardized scales to assess the sensory effects of nicotine [29, 30], the comprehensive inclusion criteria that consider both adult smokers and youth EC users in the sample, and the selection of ecologically valid FB fractions and nicotine concentrations to examine the effects of EC nicotine dimensions on product appeal. While population data indicate that self-reported use of recent ECs with NS is associated with greater symptoms of dependence among youth [34], no clinical study has been conducted to isolate the effect of EC nicotine dimensions. The study uses 10 lab-prepared, tobacco-flavored liquids varied by granular fractions of FB nicotine (5%, 25%, 45%, 65%, 85%) and concentration (20 vs. 50 mg/mL) to systematically examine EC nicotine dimensions and provide robust evidence demonstrating how to regulate EC nicotine dimensions. Ultimately, the results will help the FDA develop e-liquid product standards to reduce the uptake of ECs by youth while maintaining the possible benefits for those who smoke cigarettes.

## Supporting information

S1 File Consent form. (PDF)

S2 File Study protocol. (PDF)

S1 Table SPIRIT 2013 checklist. (PDF)
