# Supplementary material for: The sweet spot study—Developing e-liquid product standards for nicotine form and concentration to improve public health: Protocol for a randomized, double-blinded, crossover study
Source: PLoS One. 2023 Sep 12;18(9):e0291522. doi: 10.1371/journal.pone.0291522 (PMC10497122; doi:10.1371/journal.pone.0291522)
Supplement: S1 File — (PDF) [file pone.0291522.s001.pdf]

The Ohio State University Consent to Participate in Research

**Study Title:**	The impact of E-cigarette liquid nicotine form and concentration on appeal and puffing behavior
**Principal Investigator:**	Theodore L. Wagener, Ph.D.
**Sponsor:**	The Ohio State University Comprehensive Cancer Center

- **This is a consent form for research participation.** It contains important information about this study and what to expect if you decide to participate. Please consider the information carefully. Feel free to discuss the study with your friends and family and to ask questions before making your decision about whether to participate.

- **Your participation is voluntary.** You may refuse to participate in this study. If you decide to take part in the study, you may leave the study at any time. No matter what decision you make, there will be no penalty to you, and you will not lose any of your usual benefits. Your decision will not affect your future relationship with The Ohio State University. If you are a student or employee at Ohio State, your decision will not affect your grades or employment status.
- **You may or may not benefit as a result of participating in this study.** Also, as explained below, your participation may result in unintended or harmful effects for you that may be minor or may be serious depending on the nature of the research.
- **You will be provided with any new information that develops during the study that may affect your decision on whether or not to continue to participate.** If you decide to participate, you will be asked to sign this form and will receive a copy of the form. You are being asked to consider participating in this study for the reasons explained below.

Key Information About This Study

The following is a short summary to help you decide whether to participate in this study. More detailed information is listed later in this form.

You will be asked if you would like to participate in this study after you have been identified as eligible. If you choose to participate, you would be enrolled in this study. You will attend one laboratory visit, which will take up to 3 hours. The purpose of this study is to examine the influence of nicotine on vaping behavior. In this study, we are using e-liquids varying in form and concentration to help us understand how nicotine affects product appeal and vaping behavior.

If you are a female of childbearing potential, a urine pregnancy test will be performed at each visit.

You will be asked to abstain from all tobacco, nicotine, and marijuana use for at least 12 hours before you come for one laboratory visit. During this visit, we will draw blood to confirm that you have abstained from nicotine for at least 12 hours before your visit. After confirmation of a 12-hour nicotine abstinence, you will complete surveys and questionnaires regarding your sociodemographic, tobacco use history, and nicotine dependence.

You will then have the opportunity to use an e-cigarette and sample 10 different e-liquids. You will take two puffs of each of the ten e-liquid samples on the study device. E-liquid samples will be provided in random order. Between different e-liquid samples, you will rinse your mouth and spit three times with room-temperature water before sampling the next e-liquid. Each puffing session will take place in a seated position at the Ohio State University Comprehensive Cancer Center's Center for Tobacco Research.

There is no direct benefit to you by participating in this study. However, using this research, we hope the information learned will help us to understand the influence of nicotine form on vaping behavior. Potential risks include the risk of using e-cigarettes and loss of confidentiality or privacy. More information about

these risks can be found below. You will be compensated for your time and effort in this study.

1. **Why is this study being done?**

The purpose of this study is to examine the influence of nicotine form and concentration on product appeal and vaping behavior.

2. **How many people will take part in this study?**

Approximately 132 people will take part in this study.

3. **What will happen if I take part in this study?**

You will be asked if you would like to participate in this study after you have been identified as eligible. If you choose to participate, you would be enrolled in this research at Ohio State University. You will attend a single laboratory visit at the Center for Tobacco Research (directions will be provided separately). The visit will consist of a health screening, an opportunity to use the study e-cigarette with different e-liquids, and the completion of online questionnaires and surveys on a tablet. The visit will also include evaluating that you abstained from nicotine for 12 hours. Questionnaires and surveys consist of socioeconomic, tobacco history, and other evaluative questions.

If you experience any severe side effects or serious events, including an increase in preexisting illnesses while on the study, please inform the study staff immediately.

Below summarizes what activities to expect at the study visit, which will take about 3 hours:

1) Informed Consent
2) An exhaled carbon monoxide reading (eCO)
3) Urine Pregnancy Test (Female participants only)
4) Urine testing to confirm smoking/vaping status
5) Blood draw to test nicotine abstinence
6) Questionnaires (e.g., sociodemographics and tobacco use history)

7) 2-Puff Vaping Session (10-minute rest after testing each of 10 e-liquids)
8) Post-Vaping Session Evaluation Scales and Questionnaires
9) Visit Compensation ($125)

4. How long will I be in the study?

You will complete a single visit that takes about 3 hours.

5. Can I stop being in the study?

You may leave the study at any time. If you decide to stop participating in the study, there will be no penalty to you, and you will not lose any benefits to which you are otherwise entitled. Your decision will not affect your future relationship with the Ohio State University.

6. What risks, side effects, or discomforts can I expect from being in the study?

Potential risks include the risk of questionnaires, risk of loss of confidentiality, reproductive risks for women, risk of using e-cigarettes, and risk of drawing blood.

Risk of Questionnaires:
Questionnaires are non-invasive and involve minimal risk. Some people feel uncomfortable answering questions about smoking or their health. You may choose to select “Refuse to answer” as your response. Since these surveys will be administered through the University approved Research Electronic Data Capture (REDCap) system, any information collected will be in a secure database accessible only to authorized study staff.

Risk of Loss of Confidentiality and Privacy:
Every effort will be made to ensure your confidentiality will always be maintained. You will be assigned a unique study identification number (study ID). All study data will be identified by study ID only. Any electronic

information (e.g., questionnaire data, laboratory data, etc.) will only be accessible to authorized study personnel who have the necessary password(s). All computer systems will be password protected against intrusion; all network-based inter-site communications of confidential information will be encrypted. Your information will be accessible only to research staff, who are pledged to confidentiality and complete training in the ethical conduct of research (i.e., both HIPAA and CITI trainings). Identifying information will not be reported in any publication.

Reproductive Risks for Women:
If you are a female, you must not be and should not become pregnant nor breastfeed an infant while in this study. Using cigarettes or e-cigarettes while pregnant or breastfeeding may involve risks to an embryo, fetus, or infant, including birth defects that are currently unforeseeable.

If you are pregnant or suspect that you are pregnant during this study, you should immediately inform the study personnel. We will conduct a pregnancy test to ensure that you are not currently pregnant. If pregnancy is confirmed, you will be withdrawn from the study. Payment for all aspects of obstetrical, child, or related care will be your responsibility.

Risk of Using E-cigarettes:
The EC device (battery and refillable e-liquid cartridge) in our study is currently available for purchase in stores or online; however, there was no FDA evaluation of the device prior to its being commercially available. There are no known commercial e-liquids available that match the products used in this study. However, an investigational tobacco product application has been submitted to the FDA for the use of the 10 e-liquids that have been prepared. While research demonstrates that e-cigarettes are likely much less harmful than cigarettes, they are not safe. We do not know their long-term health effects. E-cigarette potential side effects are consistent with any product that contains nicotine and include nausea, headache, disrupted sleep, cough, diarrhea, flatulence, heartburn, and hiccups. You should not use e-cigarettes if you have a known allergy to propylene glycol or vegetable glycerin.

Participants should stop using these products and seek medical attention right away if any of these severe side effects occur: develop persistent indigestion; severe sore throat; irregular heartbeat or palpitations occur; severe allergic reaction (rash, hives, itching, difficulty breathing, tightness in the chest, swelling of the mouth, face, lips, or tongue); fast or irregular heartbeat; pounding in the chest; severe diarrhea, dizziness, nausea, vomiting, or weakness. Though the risk of such side effects is very low, quitting all nicotine use is ideal. If you would like to quit and/or have a quit date in mind, please let us know.

Risk of Drawing Blood:
The risks involved in drawing blood from a vein may include but are not limited to, momentary discomfort at the site of the blood draw, possible bruising, redness, and swelling around the site, bleeding at the site, feeling of dizziness or lightheadedness when the blood is drawn, nausea, and rarely, an infection at the site of the blood draw. Blood will be drawn by trained research staff/research nurses. Sterile instruments will be used, and for blood draws, the participant's skin will be cleaned with an alcohol wipe at the site of the needle stick.

7. What benefits can I expect from being in the study?

If you agree to take part in this study, there may or may not be a direct medical benefit to you. We hope that the information learned from this study will help us to understand how nicotine affects people's vaping behaviors. Future benefits to e-cigarette users include furthering scientific knowledge about the impact of nicotine on product appeal and vaping behavior.

8. What other choices do I have if I do not take part in the study?

You may choose not to participate without penalty or loss of benefits to which you are otherwise entitled.

9. Will my study-related information be kept confidential?

Efforts will be made to keep your study-related information confidential. However, there may be circumstances where this information must be released. For example, personal information regarding your participation in this study may be disclosed if required by state law.

Also, your records may be reviewed by the following groups (as applicable to the research):

- Office for Human Research Protections or other federal, state, or international regulatory agencies;
- U.S. Food and Drug Administration;
- The Ohio State University Institutional Review Board or Office of Responsible Research Practices;
- The sponsor supporting the study, their agents or study monitors; and
- Your insurance company (if charges are billed to insurance).

Authorized Ohio State staff not involved in the study may be aware that you are participating in a research study and have access to your information.

If this study is related to your medical care, your study-related information may be placed in your permanent hospital, clinic, or physician's office records. Authorized Ohio State University staff not involved in the study may be aware that you are participating in a research study and have access to your information.

If we find information that significantly impacts your health, we will share it with you. You will be provided with any new information that develops during the course of the research that may affect your decision whether or not to continue participation in the study.

A description of this clinical trial will be available on http://www.ClinicalTrials.gov, as required by U.S. law. This website will not include information that can identify you. At most, the website will include a summary of the results. You can search the website at any time.

We will work to make sure that no one sees your survey responses without approval. But, because we are using the Internet, there is a chance that someone could access your online responses without permission. In some cases, this information could be used to identify you.

Your data will be protected with a code to reduce the risk that other people can view the responses.

However, these protections do not apply in some situations. For example, we may have to release your information if a law requires us to do so, the Agency that is funding this study requests the information, or the FDA tells us to release this information. We may also use your information to conduct other scientific research as allowed by federal regulations.

Study information that has health implications may be placed in your medical record, where authorized employees may see the information. Further, authorized requests for your records (medical record release for continuity of care) may result in research-related information being released.

Please talk to your study team or contact the Office of Responsible Research Practices at 614-688-8641, if you have questions.

You may also visit the NIH website at https://grants.nih.gov/policy/humansubjects/coc.htm to learn more.

10. Will my de-identified information be used or shared for future research?

Yes, it may be used or shared with other researchers without your additional informed consent. De-identified information does not include any details that could make it possible for others to recognize you.

11. What are the costs of taking part in this study?

There is no cost to you if you participate in this study.

12. Will I be paid for taking part in this study?

By law, payments to participants are considered taxable income.

You will receive $125 as compensation for your time.

Payments will be made using the Greenphire ClinCard to increase accountability and facilitate ease of payment.

Also, rideshare services (i.e., Uber and Lyft) will be made available (within 20 miles or less of the study location) as a transportation option to and from study visits.

13. What happens if I am injured because I took part in this study?

If you suffer an injury from participating in this study, you should notify the PI immediately, who will help determine if you should obtain medical treatment at Ohio State University Wexner Medical Center.

The cost for this treatment will be billed to you or your medical or hospital insurance. The Ohio State University has no funds set aside for the payment of health care expenses for this study.

14. What are my rights if I take part in this study?

If you choose to participate in the study, you may discontinue participation at any time without penalty or loss of benefits. By signing this form, you do not give up any personal legal rights you may have as a participant in this study.

You will be provided with any new information that develops during the course of the research that may affect your decision whether or not to continue participating in the study.

You may refuse to participate in this study without penalty or loss of benefits to which you are otherwise entitled.

An Institutional Review Board responsible for human subjects research at The Ohio State University reviewed this research project and found it acceptable, according to applicable state and federal regulations and University policies designed to protect the rights and welfare of research participants.

15. Who can answer my questions about the study?

For questions, concerns, or complaints about the study, you may contact study staff at 1-844-744-2447.

For questions about your rights as a participant in this study or to discuss other study-related concerns or complaints with someone who is not part of the research team, you may contact the Office of Responsible Research Practices at 1-800-678-6251.

If you are injured as a result of participating in this study or for questions about a study-related injury, you may contact Dr. Theodore L. Wagener at 844-744-2447.

If you change your mind about taking part in this study, you can contact study staff by phone at 1-844-744-2447.

Signing the consent form

I have read this form, and I am aware that I am being asked to participate in a research study. I have had the opportunity to ask questions and have had them answered to my satisfaction. I voluntarily agree to participate in this study.

I am not giving up any legal rights by signing this form. I will be given a copy of this form.

Subject Name (Printed): ____________________________

Subject Signature: ____________________________Date: ________Time:_______ am pm

Investigator/Research Staff

I have explained the research to the participant or his/her representative before requesting the signature(s) above. There are no blanks in this document. A copy of this form has been given to the participant or his/her representative.

Printed Name of Person Obtaining Consent: _____________________________________

Signature of Person Obtaining Consent: ____

Date: ____________ Time: ________ am pm
