# Supplementary material for: The sweet spot study—Developing e-liquid product standards for nicotine form and concentration to improve public health: Protocol for a randomized, double-blinded, crossover study
Source: PLoS One. 2023 Sep 12;18(9):e0291522. doi: 10.1371/journal.pone.0291522 (PMC10497122; doi:10.1371/journal.pone.0291522)
Supplement: S2 File — (PDF) [file pone.0291522.s002.pdf]

IRB Protocol Number: 2022C0207
IRB Initial Approval Date: 4/10/2023
Version: 1.0

RESEARCH PROTOCOL

Title: Nicotine Concentration and Forms: Differential Appeal to Smokers versus Non-smokers (Sweet Spot Study)

Contents

I. Summary, Background, and Objectives ... 2
- A. Summary ... 2
- B. Background ... 2
- C. Objectives ... 3

II. Research Methods & Activities ... 3
- A. Design Overview ... 3
- B. Study Procedures ... 3
- C. Recruitment ... 4
  - 1) Number of Participants ... 4
  - 2) Participant Population ... 4
  - 3) Participant Identification ... 5
  - 4) Incentives to Participate ... 5
- D. Measurement ... 5
  - 1) Self-report measures ... 5
  - 2) E-cigarette Device and E-liquids ... 6
- E. Data Analysis ... 6

III. Data and Safety Monitoring Plan ... 7
- A. Informed Consent Process ... 7
- B. Privacy of Participants ... 7
- C. Confidentiality of Data ... 7

IV. Human Participants ... 7
- A. Reasonably Anticipated Benefits ... 7
- B. Potential Risks ... 7
- C. Protections Against Risk ... 8
- D. Assessment of Risks & Benefits ... 8

V. Bibliography ... 8

I. Summary, Background, and Objectives

A. Summary

The tobacco industry frequently manipulates nicotine in cigarettes to promote smoking. Similarly, e-cigarette (EC) manufacturers manipulate nicotine dimensions, including concentration and form. These manipulations have increased palatability and nicotine delivery, leading to increased appeal and addictiveness, particularly among young people. While ECs pose less risks to smokers who completely switch from cigarettes to ECs, EC use among young people is alarming. Regulating nicotine can potentially make products unappealing to young people while still providing smokers with a less harmful alternative with sufficient satisfaction. The overall aim of the proposed study is to determine if an EC product standard requiring a minimum level of free-base nicotine (nicotine form) would reduce the appeal of ECs for young adults but not older adult smokers who are more accustomed to the harshness of free-base nicotine. A single-visit, double-blind, randomized crossover design will be used, where young adult exclusive EC users with minimal or no history of smoking and older adult smokers complete EC puffing sessions and assess outcomes of appeal and sensory experience, using e-liquids that differ by 2 nicotine concentrations and varying free-base nicotine fractions. The study will be the first to examine the impact of nicotine dimensions on EC abuse liability and determine whether regulations limiting free-base nicotine fraction in EC liquids are appropriate for public health. Results will inform marketing and product regulations that reduce appeal in young people who do not smoke but not in adult tobacco users.

B. Background

E-cigarettes (ECs) provide a less harmful substitute for smokers who completely switch, but they pose significant risks to young non-smokers. Evidence is conclusive that completely substituting ECs for cigarettes reduces users' exposure to tobacco-specific toxicants and carcinogens.[1,2] While evidence from population studies is mixed on the effectiveness of ECs to help smoking cessation,[3] frequent use of ECs[4] and use of newer EC types with improved nicotine delivery is associated with smoking cessation.[5] However, EC use is prevalent among youth,[6] which is concerning because ECs cause nicotine addiction[7] and increase risk of smoking.[8,9] The fact that ECs can benefit smokers but pose potential risks among young non-smokers challenges the Food and Drug Administration (FDA) to regulate ECs in a way that allow ECs to remain as appealing substitutes for current smokers while reducing their appeal to young people. Manipulating nicotine dimensions is potentially an effective regulatory strategy to increase the appeal of ECs to smokers while reducing their appeal to young people. EC manufacturers, for instance, manipulate nicotine to increase palatability and nicotine delivery, maximizing their appeal and addictiveness. ECs have evolved from using EC liquids with unprotonated 'free-base' nicotine to protonated 'nicotine salts.' The use of nicotine salts made EC aerosols easier to inhale especially for nicotine naïve users, allowing the EC industry to significantly increase nicotine concentration.[10] Easier inhalation for cigarettes was associated with higher abuse liability, but more studies are needed to determine the effects of manipulating EC nicotine dimensions, including nicotine form, on product appeal and abuse liability. Such studies will help FDA determine how to effectively regulate nicotine to benefit public health.

C. Objectives

The proposed study aims to assess the effect of e-liquids with nicotine varying in freebase (FB) levels (5, 25, 45, 65, 85%) and concentrations (20 or 50 mg/mL) on appeal among young adult EC users with minimal/no history of smoking (n=66) and older adult smokers (n=66), using a single-visit, double-blind, randomized crossover trial design. The study will determine freebase nicotine fractions that minimally appeal to young adult EC users but sufficiently appeal to older adult smokers, across ecologically valid nicotine concentrations. The study will test a hypothesis that increasing FB levels (i.e., decreasing nicotine salts) will make ECs less appealing to young adult EC users but not for older adult smokers, especially at higher nicotine concentration.

II. Research Methods & Activities

A. Design Overview

In the proposed study, a sample of young adult (21-24 years) exclusive EC users with no or minimal history of smoking (n=66) and older adult (25-50 years) smokers (n=66) will complete a single-visit, double-blinded, within-subject randomized crossover trial. Participants will vape e-liquids containing varying freebase (FB) nicotine fractions (5, 25, 45, 65, 85%) at 20 or 50 mg/mL concentrations. The FB fractions were selected to cover a wide range with the lower end consistent with JUUL and disposable pod-based ECs that are popular among youth[11] and the higher end resembling early ECs that were FB nicotine-based and less appealing to young people. Each of the 10 conditions will be tested in a 2-puff session, followed by rating the appeal and sensory attributes, with a 10-minute washout period between conditions. Previous studies used a similar study design to examine the appeal of EC flavors, nicotine concentrations, and interactive effects of flavor and nicotine.[12-15] For example, in a study of young adult current EC users,[12] the sensory appeal of ECs decreased as nicotine concentration increased but was not affected by flavoring. Another study of current EC users or smokers reported higher ratings of appeal for salt nicotine vs. free-base nicotine across different flavors, and salt nicotine decreased perceived harshness more among never smokers than ever smokers.[15] While these studies shed light on the effect of nicotine salts and concentration on the sensory experience of EC use, their design was limited in ability to evaluate the effect of granular fractions of nicotine salts. This study will fill the gap in the literature by examining the effect of five free-base levels of nicotine at two nicotine concentration levels and determining the ‘sweet spot’ level of free-base nicotine that can be tolerated by smokers but not by non-smokers.

B. Study Procedures

Participants will be screened initially through an online screener, which will be linked to our study advertisement and administered using REDCap. Eligible participants interested in participating will be scheduled for the study visit via phone or e-mail. Participants will be instructed that they must remain nicotine abstinent for 12 hours before their visit and will receive a reminder the evening before.

Each participant will make one laboratory visit, which will take up to 3 hours. After obtaining their informed consent at the study visit, eligibility to participate in vaping sessions will be determined. Smoking status will be biochemically confirmed via an exhaled carbon monoxide reading (eCO) of ≥4ppm and a urine cotinine testing kit, and exclusive EC use status will be confirmed with eCO (<4ppm) and the cotinine test, and cross-validated against self-reports.[16] A urine pregnancy test will be administered if the participant’s biological sex is female. Urine sample will be collected and analyzed for

the level of total NNAL, a metabolite of NNK that is found only in cured tobacco and is present at extremely low levels in EC users,[16] using validated methods[17-20] following study visits. As a semi-bogus pipeline procedure[21] to improve the truthfulness of self-reports, participants will be told that the urine sample will be analyzed at the time of the visit to confirm current smoking in smokers (≥0.15 pmol/mL urine total NNAL) and no concurrent use of combustible and smokeless tobacco use among young adult exclusive EC users (<0.15 pmol/mL). Those who do not meet the criteria will be replaced post hoc with additional participants. Blood sample will also be collected as a semi-bogus pipeline procedure to confirm nicotine abstinence. Participants will be told that their abstinence will be confirmed at the time of visit via blood plasma nicotine analysis (this is a semi-bogus pipeline as 3mL venous blood sample will not be analyzed in real-time but collected for later analysis and those with baseline plasma nicotine levels >=3ng/mL will be replaced post hoc with another participant).

Vaping sessions will take place after collecting biospecimens and completing questionnaires on sociodemographics, tobacco use history, and dependence on ECs and cigarettes. Guided by study staff, participants will take two puffs of each of the 10 study conditions (5 freebase nicotine fractions x 2 nicotine concentrations) using the study EC device, followed by a 10-minute washout period between conditions. During the washout period, participants will be instructed to rinse their mouths and spit three times with room-temperature water before sampling the next preparation to minimize carryover effects. The washout period was deemed sufficient based on an arterial study showing a return to baseline plasma nicotine levels 40 minutes following a 10-puff vaping session (hence, 4 minutes are needed for nicotine levels to return to baseline after each puff).[22] Participants will be provided pre-filled and weighed e-liquid pods in random order. Participants will be instructed to inhale for at least 1 second for each puff, but other puffing parameters (i.e., flow rate, maximum puff duration, puff volume, rate of exhalation) will not be controlled.

After each condition, participants will complete General Labeled Magnitude Scale (gLMS)[14] and Labeled Hedonic Scale[23] to rate the degree of liking or disliking of sensations and the strength of sensory attributes of e-liquids including throat hit, smoothness, harshness, sweetness, and bitterness. Participants will also rate overall acceptance on a tobacco industry-designed thermometer scale (from 0 “the very worst” to 100 “the very best”, with 50 indicating “indifferent”).[24] Drug Effects/Liking Questionnaire,[25] the modified Cigarette Evaluation Questionnaire (mCEQ), and the adapted version of behavioral intentions for continued use (from 1 “extremely unlikely” to 7 “extremely likely”)[26] will also be collected to assess subjective responses to ECs (e.g., reward, satisfaction).[27]

All study visits will take place at the OSUCCC’s Center for Tobacco Research, where participants will complete a consent form, screening procedures, vaping sessions, and online questionnaires and surveys on a tablet.

C. Recruitment

1) Number of Participants

A total of 132 participants will be recruited, including young adult e-cigarette (EC) users with minimal or no history of smoking (n=66) and older adult smokers (n=66).

2) Participant Population

Participants who meet the following eligibility criteria will be included in the study. Inclusion criteria: 1) current exclusive young adult EC user (daily use of ≥ 1 vaping bout, defined as taking around 15 puffs or vaping for around 10 minutes) for at least the past 3 months (confirmed by cotinine test kit) between 21-24 years old with no/minimal history of smoking cigarettes (<10 cigarettes in entire life), or

current older adult smoker (daily use, ≥100 cigarettes in entire life) aged 25-50 years not currently engaged in smoking cessation activities; 2) willing to abstain from all nicotine product and marijuana use for at least 12 hours before the study visit; and 3) read and speak English. Exclusion criteria: 1) currently attempting to quit nicotine or tobacco products; 2) currently pregnant (will be verified with urine pregnancy test), planning to become pregnant, or breastfeeding; 3) use of nicotine products other than ECs or cigarettes (use of ≥ 10 traditional cigars, cigarillos, or filtered cigars in entire life; use of smokeless tobacco products ≥ 10 times in entire life; or hookah in the last 30 days); 4) self-reported diagnosis of lung disease including asthma, cystic fibrosis, or chronic obstructive pulmonary disease; and 5) self-reported new or unstable cardiovascular disease diagnosed within the past 12 months. Upon completion of the study, we will provide participants with information on free tobacco dependence treatment through the Ohio Tobacco Quitline.

3) Participant Identification

Participants will be recruited from the general community via targeted internet and social media advertisements as well as flyers and word-of-mouth advertising in the Columbus metropolitan area. Recruitment will be conducted in collaboration with the OSUCCC Social Media team, and our marketing partner, Eclectic. Eclectic is a full-service marketing firm that will provide advertising copy development, strategy (e.g., targeting variables, rotation plans), and management of ads through various platforms. Advertisements will be spread across various online and social media outlets. Over the last 5 years, our team has worked with Eclectic to coordinate the recruitment of other studies of similar design as the proposed with outstanding success.

4) Incentives to Participate

Participants will receive $125 for the prolonged lab visit (~3 hours). Payments will be made in person using the Greenphire ClinCard to increase accountability and facilitate ease of payment. We will also facilitate study visits by offering evening and weekend appointments, maintaining multiple avenues of contact (call, text, email, and mail), and multiple methods to reduce participant burden (e.g., reducing the length of visits, rideshare, etc.). The cost of rideshare will be provided to participants who live within 20 miles of OSUCCC CTR, except for those who take other forms of transportation.

D. Measurement

1) Self-report measures

Participants will complete questionnaires on sociodemographics, tobacco use history, and EC and cigarette dependence upon enrollment. Sociodemographic measures will assess participant age, sex (male/female), marital status, ethnicity, employment status, years of education, and annual household income. Tobacco use history will assess years of tobacco use, age of first use, tobacco type, brand, frequency, quantity, and duration of using all nicotine products including cigars, cigarillos, little cigars, pipe tobacco, chewing tobacco, snuff, snus, EC/vape/mod/APV/e-hookah, and hookah tobacco. Cigarette Dependence will be measured with the 10-item Penn State Cigarette Dependence Index[28] and the 12-item Cigarette Dependence Scale.[29] EC Dependence will be measured with both a modified 12-item EC Dependence Scale and the Penn State EC Dependence Index.[29,30]

After taking two puffs of each of the 10 study conditions (5 freebase nicotine fractions x 2 nicotine concentrations), participants will complete the General Labeled Magnitude Scale (gLMS)[14] and Labeled Hedonic Scale[23] to rate the liking or disliking of sensation and the strength of sensory attributes (throat hit, smoothness, harshness, sweetness, bitterness), and a tobacco industry-designed thermometer rating scale of overall acceptance (from 0 “the very worst” to 100 “the very best”, with 50

indicating "indifferent").[24] Participants will also complete the Drug Effects/Liking Questionnaire which will assess the desire and liking of study products, positive and negative effects, and perceived strength and effectiveness.[25] The modified Cigarette Evaluation Questionnaire (mCEQ)[27] and behavioral intention to continued use[26] will also assess subjective responses to ECs (e.g., reward, satisfaction).

2) E-cigarette Device and E-liquids

Study EC Device: JUUL will be used as EC device by all participants during sessions, a refillable pod-based device.

Study E-liquids: As there are no known commercial e-liquids that match the requirements for the proposed study, we will prepare the 10 e-liquids with different levels of freebase nicotine. The test liquids will be prepared using USP-grade nicotine, benzoic acid, glycerol, and propylene glycol, and flavored with tobacco flavoring. We will submit an ITP application to the US FDA to receive approval for the e-liquids. The study team has successfully completed ITP applications for other tobacco products, including the production of e-liquids (e.g., U01 DA045537; U01 DA045530; R01 CA209961; R01 CA229306).

E. Data Analysis

All statistical analyses will be performed using SAS 9.4 (SAS Institute, Cary, NC). Demographic and other participant characteristics will be summarized by the study arm. Continuous variables will be presented as mean ± standard deviation and compared with t-tests while categorical variables will be presented as counts and proportions and compared with chi-squared tests. We will apply transformations to normalize the distribution and stabilize the variance of the residuals where appropriate.

The primary goal of the study is to identify a nicotine fraction limit that is appealing to smokers with a minimal appeal to young adult EC users. Generalized linear mixed models will be employed for measures of appeal (e.g., industry thermometer rating and sensory appeal metrics). Models will include demographic and smoking history as covariables, random effects for subject, main effects for user group (smokers vs. EC use), FB fraction (numeric), and concentration (categorical), and interaction effects between smoking group and each of the condition parameter variables (i.e., FB fraction and concentration). A significant interaction between smoking group and a condition parameter variable would indicate that the variable differentially affects appeal between the two user groups. Because of multiple measurements tested, we will assume a significance threshold of p=0.01. Conservatively, considering 120 participants, a correlation of 0.5, and alpha=0.01, a two-sided paired t-test achieves 80% power to detect an effect size of 0.32 standard deviations of the measurement for the concentration main effect. For FB fraction, with 120 subjects, the model achieves 80% power to detect a slope of 0.31, considering alpha=0.01. We estimate the power for the categorical interactions by pairwise comparisons of the two interacting groups (with two 2-level variables there are a total of 4 groups) and adjust the p-value to account for the 6 possible pairwise comparisons (0.01/6=0.0017=p). With 30 samples per group, a two-sided, two-sample t-test achieves over 80% power to detect a 1.7-fold difference between any of the two groups in the interaction term, considering alpha=0.0017 and coefficient of variation=0.5. For interactions with FB fraction, group sizes of 60 achieve 80% power to detect a difference in slopes of 0.43, considering alpha=0.01. Interactions between condition parameter variables will also be explored. Since our goal is to find combinations of the condition parameter that are appealing to smokers but not young adult EC users, we will compare condition combinations directly to identify the condition parameter combinations that result in the largest difference in appeal between the two user groups. Carryover effects will be explored by estimating the interaction of a given condition with the preceding treatment and period effects tested by the interaction of treatment with order.

III. Data and Safety Monitoring Plan

A. Informed Consent Process

Informed consent (including a description of the nature, purpose, risks, and benefits of the study) will take place through both oral and written explanation of the study. A legally valid signature will be obtained via REDCap. The voluntary nature of the study and the participant's right to withdraw at any time will be stressed during the consent process; a copy of the informed consent will be provided to the participant in written form at the time of consent for them to keep via e-mail. Informed consent will be collected in person by IRB-approved study personnel. The Ohio State University Institutional Review Board will approve recruitment scripts and materials, consent forms, and all study procedures. All participants will provide written consent before any study data are collected.

B. Privacy of Participants

To ensure privacy study visits will take place in a secure laboratory setting at the Center for Tobacco Research where access is limited to study personnel. Researchers will work with only one participant at a time to protect the privacy of participants. Given that participants may find questions about EC use intrusive, they will be informed about the nature of questions in advance.

C. Confidentiality of Data

Study subjects' confidentiality will be always maintained. Participants will be assigned a unique study identification number (study ID). All study data will be identified by study ID only. Tablets will be supplied by Ohio State and used to enter electronic data. Electronic data (e.g., questionnaire data and laboratory data) will be obtained directly from participants. The electronic data will be entered, stored, and secured through REDCap and managed by Ohio State University, which will only be accessible to authorized study personnel who have the necessary password(s). All computer systems will be password protected against intrusion; all network-based inter-site communications of confidential information will be encrypted. Access to computer-stored information will require simultaneous knowledge of the data format, computer language, file name and passwords. Participant information will be accessible only to research staff, who are pledged to confidentiality and complete training in the ethical conduct of research (i.e., both HIPAA and CITI trainings). Physical materials will be stored in a locked file cabinet, locked office or suite at the Center for Tobacco Research, 3650 Olentangy River Rd, Columbus, OH 43214 only.

IV. Human Participants

A. Reasonably Anticipated Benefits

There may or may not be direct medical benefits to individual participants. The information learned from this study will advance scientific knowledge about the impact of nicotine dimensions (e.g., freebase nicotine fractions) on product appeal and vaping behaviors. Such knowledge will inform future regulatory actions such as requiring a minimal level of freebase nicotine fractions, which may offer health benefits to young adult exclusive EC users and older adult smokers.

B. Potential Risks

As the research protocol calls for current EC users and adult smokers to use a study e-cigarette, the participants are already smoking or using ECs and will only be asked to use what will be a fully characterized EC device and e-liquids for which an FDA ITP will be submitted and "approved" (i.e., receive an FDA letter of "no concern"). The risk of side effects and adverse events following using ECs

are very low. These products are sold online, and at EC specialty stores and convenience stores nationwide, without a prescription. Lab studies of toxin exposure suggest that ECs incur no greater risk to health than conventional cigarettes. Indeed, ECs generally show lower levels of harmful and potentially harmful constituents. To date, EC studies discussing adverse events report mild and tolerable side effects, such as mouth/throat irritation, cough, and headache, that generally resolved completely over time with continued use. In previous randomized clinical trials, no serious adverse events were reported and the EC group and the nicotine patch group had comparable levels of adverse events in two studies, with the most common being mouth irritation, throat irritation, dry cough and headache. Questionnaires, urine, and exhaled breath procedures are all non-invasive and involve minimal risk to study participants. The risks involved in drawing blood from a vein may include, but are not limited to, momentary discomfort at the site of the blood draw, possible bruising, redness, and swelling around the site, bleeding at the site, feeling of dizziness or lightheadedness when the blood is drawn, nausea, and rarely, an infection at the site of the blood draw.

C. Protections Against Risk

All participants will be screened for general medical precautions (pregnancy, cardiovascular disease) and monitored for adverse events during the study period. Any serious adverse events will be reported to the study's PI and then to the Ohio State University IRB. Participants who have a serious adverse event or have a cardiovascular or pulmonary event during the study will be withdrawn from the study. Blood will be drawn by trained research staff/research nurse. IVs will be placed by an RN or LPN. Sterile instruments will be used, and for blood draws, the participant's skin will be cleaned with an alcohol wipe at the site of the needle stick. Following the completion of the study, we will encourage all participants to quit their use of ECs and they will be provided the number to the Ohio Tobacco Quit Line (1-900-784-8669).

D. Assessment of Risks & Benefits

In general, this study will provide a benefit to future EC users and smokers as the findings will be reported to public health officials and the FDA to inform policy, for encouraging smoking cessation, identifying abuse liability effects, and reducing toxicity (if any). All participants will be encouraged to quit smoking and/or using ECs at the completion of the study. Adequate protections are in place in the event of unlikely and mild risks to study participation. Research results will not be returned to participants. Overall, it is expected that the potential benefits to participants in the proposed study outweigh the potential risks.

V. Bibliography

1. National Academies of Sciences, Engineering, Medicine. *Public Health Consequences of E-Cigarettes*. The National Academies Press; 2018:774.
2. Dai H, Benowitz NL, Achutan C, Farazi PA, Degarege A, Khan AS. Exposure to Toxicants Associated With Use and Transitions Between Cigarettes, e-Cigarettes, and No Tobacco. *JAMA Network Open*. 2022;5(2):e2147891-e2147891. doi:10.1001/jamanetworkopen.2021.47891
3. Hartmann-Boyce J, McRobbie H, Butler AR, et al. Electronic cigarettes for smoking cessation. *Cochrane Database of Systematic Reviews*. 2021;(9)doi:10.1002/14651858.CD010216.pub6
4. Kalkhoran S, Chang Y, Rigotti NA. Electronic Cigarette Use and Cigarette Abstinence Over 2 Years Among U.S. Smokers in the Population Assessment of Tobacco and Health Study. *Nicotine & Tobacco Research*. 2019;22(5):728-733. doi:10.1093/ntr/ntz114

5. Glasser AM, Vojjala M, Cantrell J, et al. Patterns of E-cigarette Use and Subsequent Cigarette Smoking Cessation Over 2 Years (2013/2014–2015/2016) in the Population Assessment of Tobacco and Health Study. *Nicotine & Tobacco Research*. 2020;23(4):669-677. doi:10.1093/ntr/ntaa182
6. Park-Lee E, Ren C, Sawdey MDG, Andrea S, Cornelius M, J, Ahmed, Cullen KA. E-Cigarette Use Among Middle and High School Students — National Youth Tobacco Survey, United States, 2021. *Morbidity and Mortality Weekly Report*. 2022;70(39):1387.
7. Vogel EA, Prochaska JJ, Ramo DE, Andres J, Rubinstein ML. Adolescents' E-Cigarette Use: Increases in Frequency, Dependence, and Nicotine Exposure Over 12 Months. *Journal of Adolescent Health*. 2019/06/01/ 2019;64(6):770-775. doi:https://doi.org/10.1016/j.jadohealth.2019.02.019
8. O'Brien D, Long J, Quigley J, Lee C, McCarthy A, Kavanagh P. Association between electronic cigarette use and tobacco cigarette smoking initiation in adolescents: a systematic review and meta-analysis. *BMC Public Health*. 2021/06/03 2021;21(1):954. doi:10.1186/s12889-021-10935-1
9. Loukas A, Marti CN, Cooper M, Pasch KE, Perry CL. Exclusive e-cigarette use predicts cigarette initiation among college students. *Addictive Behaviors*. 2018/01/01/ 2018;76:343-347. doi:https://doi.org/10.1016/j.addbeh.2017.08.023
10. Barrington-Trimis JL, Leventhal AM. Adolescents' Use of "Pod Mod" E-Cigarettes — Urgent Concerns. *New England Journal of Medicine*. 2018;379(12):1099-1102. doi:10.1056/NEJMp1805758
11. Goniewicz ML, Boykan R, Messina CR, Eliscu A, Tolentino J. High exposure to nicotine among adolescents who use Juul and other vape pod systems ('pods'). *Tobacco Control*. 2019;28(6):676-677. doi:10.1136/tobaccocontrol-2018-054565
12. Pullicin AJ, Kim H, Brinkman MC, Buehler SS, Clark PI, Lim J. Impacts of Nicotine and Flavoring on the Sensory Perception of E-Cigarette Aerosol. *Nicotine & Tobacco Research*. 2019;22(5):806-813. doi:10.1093/ntr/ntz058
13. Leventhal A, Cho J, Barrington-Trimis J, Pang R, Schiff S, Kirkpatrick M. Sensory attributes of e-cigarette flavours and nicotine as mediators of interproduct differences in appeal among young adults. *Tobacco Control*. 2020;29(6):679-686. doi:10.1136/tobaccocontrol-2019-055172
14. Rosbrook K, Green BG. Sensory Effects of Menthol and Nicotine in an E-Cigarette. *Nicotine & Tobacco Research*. 2016;18(7):1588-1595. doi:10.1093/ntr/ntw019
15. Leventhal AM, Madden DR, Peraza N, et al. Effect of Exposure to e-Cigarettes With Salt vs Free-Base Nicotine on the Appeal and Sensory Experience of Vaping: A Randomized Clinical Trial. *JAMA Network Open*. 2021;4(1):e2032757-e2032757. doi:10.1001/jamanetworkopen.2020.32757
16. Benowitz NL, Bernert JT, Foulds J, et al. Biochemical Verification of Tobacco Use and Abstinence: 2019 Update. *Nicotine & Tobacco Research*. 2019;22(7):1086-1097. doi:10.1093/ntr/ntz132
17. Stepanov I, Carmella SG, Briggs A, et al. Presence of the Carcinogen N′-Nitrosonornicotine in the Urine of Some Users of Oral Nicotine Replacement Therapy Products. *Cancer Research*. 2009;69(21):8236-8240. doi:10.1158/0008-5472.Can-09-1084
18. Stepanov I, Carmella SG, Han S, et al. Evidence for endogenous formation of N′-nitrosonornicotine in some long-term nicotine patch users. *Nicotine & Tobacco Research*. 2009;11(1):99-105. doi:10.1093/ntr/ntn004
19. Stepanov I, Upadhyaya P, Carmella SG, et al. Extensive Metabolic Activation of the Tobacco-Specific Carcinogen 4-(Methylnitrosamino)-1-(3-Pyridyl)-1-Butanone in Smokers. *Cancer Epidemiology, Biomarkers & Prevention*. 2008;17(7):1764-1773. doi:10.1158/1055-9965.Epi-07-2844
20. Carmella SG, Han S, Fristad A, Yang Y, Hecht SS. Analysis of Total 4-(Methylnitrosamino)-1-(3-Pyridyl)-1-Butanol (NNAL) in Human Urine. *Cancer Epidemiology, Biomarkers & Prevention*. 2003;12(11):1257-1261.

21. Roese NJ. Twenty years of bogus pipeline research: A critical review and meta-analysis. *Psychological bulletin*. 1993 1993;114(2):363-375. doi:10.1037/0033-2909.114.2.363
22. Baldassarri SR, Hillmer AT, Anderson JM, et al. Use of Electronic Cigarettes Leads to Significant Beta2-Nicotinic Acetylcholine Receptor Occupancy: Evidence From a PET Imaging Study. *Nicotine & Tobacco Research*. 2017;20(4):425-433. doi:10.1093/ntr/ntx091
23. Lim J, Wood A, Green BG. Derivation and Evaluation of a Labeled Hedonic Scale. *Chemical Senses*. 2009;34(9):739-751. doi:10.1093/chemse/bjp054
24. Rees VW, Kreslake JM, Cummings KM, et al. Assessing consumer responses to potential reduced-exposure tobacco products: a review of tobacco industry and independent research methods. *Cancer Epidemiol Biomarkers Prev*. Dec 2009;18(12):3225-40. doi:10.1158/1055-9965.Epi-09-0946
25. Zacny JP, Conley K, Marks S. Comparing the Subjective, Psychomotor and Physiological Effects of Intravenous Nalbuphine and Morphine in Healthy Volunteers. *Journal of Pharmacology and Experimental Therapeutics*. 1997;280(3):1159-1169.
26. Wagener TL, Leavens ELS, Mehta T, et al. Impact of flavors and humectants on waterpipe tobacco smoking topography, subjective effects, toxicant exposure and intentions for continued use. *Tobacco Control*. 2021;30(4):366-372. doi:10.1136/tobaccocontrol-2019-055509
27. Cappelleri JC, Bushmakin AG, Baker CL, Merikle E, Olufade AO, Gilbert DG. Confirmatory factor analyses and reliability of the modified cigarette evaluation questionnaire. *Addictive Behaviors*. 2007/05/01/ 2007;32(5):912-923. doi:https://doi.org/10.1016/j.addbeh.2006.06.028
28. Foulds J, Veldheer S, Yingst J, et al. Development of a Questionnaire for Assessing Dependence on Electronic Cigarettes Among a Large Sample of Ex-Smoking E-cigarette Users. *Nicotine & Tobacco Research*. 2014;17(2):186-192. doi:10.1093/ntr/ntu204
29. Etter J-F, Le Houezec J, Perneger TV. A Self-Administered Questionnaire to Measure Dependence on Cigarettes: The Cigarette Dependence Scale. *Neuropsychopharmacology*. 2003/02/01 2003;28(2):359-370. doi:10.1038/sj.npp.1300030
30. Park E, Kwon M, Chacko T, et al. Instruments to measure e-cigarette related constructs: a systematic review. *BMC Public Health*. 2022/06/07 2022;22(1):1135. doi:10.1186/s12889-022-13510-4
